# A Label-Free Gold Nanoparticles-Based Optical Aptasensor for the Detection of Retinol Binding Protein 4

**DOI:** 10.3390/bios12121061

**Published:** 2022-11-22

**Authors:** Koena L. Moabelo, Teresa M. Lerga, Miriam Jauset-Rubio, Nicole R. S. Sibuyi, Ciara K. O’Sullivan, Mervin Meyer, Abram M. Madiehe

**Affiliations:** 1 Nanobiotechnology Research Group, Department of Biotechnology, University of Western Cape, Bellville 7535, South Africa; 2 Department of Science and Innovation (DSI)/Mintek Nanotechnology Innovation Centre, Biolabels Research Node, Department of Biotechnology, University of Western Cape, Bellville 7535, South Africa; 3 Interfibio Research Group, Departament d’Enginyeria Quimica, Universitat Rovira i Virgili, Avinguda Països Catalans 26, 43007 Tarragona, Spain

**Keywords:** retinol-binding protein 4, type 2 diabetes mellitus, aptasensor, colorimetric, gold nanoparticles, diagnosis, biosensing

## Abstract

Retinol-binding protein 4 (RBP4) has been implicated in insulin resistance in rodents and humans with obesity and T2DM, making it a potential biomarker for the early diagnosis of T2DM. However, diagnostic tools for low-level detection of RBP4 are still lagging behind. Therefore, there is an urgent need for the development of T2DM diagnostics that are rapid, cost-effective and that can be used at the point-of-care (POC). Recently, nano-enabled biosensors integrating highly selective optical detection techniques and specificity of aptamers have been widely developed for the rapid detection of various targets. This study reports on the development of a rapid gold nanoparticles (AuNPs)-based aptasensor for the detection of RBP4. The retinol-binding protein aptamer (RBP-A) is adsorbed on the surface of the AuNPs through van der Waals and hydrophobic interactions, stabilizing the AuNPs against sodium chloride (NaCl)-induced aggregation. Upon the addition of RBP4, the RBP-A binds to RBP4 and detaches from the surface of the AuNPs, leaving the AuNPs unprotected. Addition of NaCl causes aggregation of AuNPs, leading to a visible colour change of the AuNPs solution from ruby red to purple/blue. The test result was available within 5 min and the assay had a limit of detection of 90.76 ± 2.81 nM. This study demonstrates the successful development of a simple yet effective, specific, and colorimetric rapid assay for RBP4 detection.

## 1. Introduction

Type 2 diabetes mellitus (T2DM) is a chronic metabolic disease with debilitating effects on human health. It is a growing epidemic, that affects over 536.6 million adults worldwide [1]. If undiagnosed or untreated, T2DM can lead to microvascular (retinopathy, neuropathy and nephropathy) and macrovascular (stroke and acute coronary syndrome) complications [2]. Many of these complications can go un-noticed for years and are often subclinical at the onset, making the early diagnosis of T2DM difficult [3]. Therefore, an urgent need to develop novel and early diagnostic strategies to address this epidemic is warranted.

Retinol-binding protein 4 (RBP4), an adipokine responsible for obesity-induced insulin resistance, has been identified as one of the reliable biomarkers for the early diagnosis of T2DM [4]. RBP4 binds to retinol and transports it in the blood stream to the liver. Elevated levels of RBP4 have been found in insulin-resistant mice and humans with obesity and T2DM, causing dysfunctions in the production of glucose transporter 4 (GLUT4) and consequently leading to a failure of glucose uptake from the blood. This suggests that RBP4 could serve as a potential serological biomarker for the early diagnosis of T2DM [5,6,7,8,9]. 

The detection of RBP4 is usually performed through different biotechnological assays, such as enzyme-linked immunosorbent assay (ELISA) and Western blot [10,11,12], which rely on the interaction between an antibody and an analyte. However, the use of antibodies in diagnostic assays have several inherent limitations such as high production costs, instability, and poor specificity, and their use requires longer incubation periods and different substrates for detection [13]. Recently, the use of aptamers in diagnostic assays have been shown to alleviate these limitations. Aptamers, which are single-stranded deoxyribonucleic acid (DNA) or ribonucleic acid (RNA) molecules that bind with high affinity and specificity to their target molecules, exhibit significant advantages relative to antibodies in terms of their high specificity, selectivity, low molecular weight, and ease of production [13,14,15]. Furthermore, aptamers present a more favourable and desirable molecular recognition element (MRE) and have been employed in the development of various sensors for diagnostic purposes [16,17,18,19,20].

Lee et al. reported on the development of the first RBP-aptamer (RBP-A) and its application in a surface plasmon resonance (SPR) biosensor for the detection of RBP4 in serum. The SPR biosensor specifically detected RBP4 and had a limit of detection of 75 nM (1.58 µg/mL), which is sufficiently sensitive to probe for RBP4 in the serum of people who are at risk of developing T2DM [21]. For instance, it has been reported that the detection range of RBP4 in normal individuals is 23.0–24.3 µg/mL (1–1.06 µM) and ranges from 24.9–50.3 µg/mL (1.08–2.187 µM) in individuals who are diagnosed with T2DM [22]. Using the same aptamer, an enzyme-linked antibody-aptamer sandwich (ELAAS) method was developed for more convenient and sensitive detection of RBP4. Compared to the SPR-based biosensor, this method was between 20 and 68 times more sensitive [23]; however, the assay involved multiple incubation and washing steps, making it impossible for it to be applied in point-of-care testing (PoCT). Torabi et al. developed an ultrasensitive chemiluminescent aptasensor for the detection of RBP4 with an LOD of 951 fg/mL [24]. Similarly to the ELAAS, this assay involved multiple washing and incubation steps, rendering it unsuitable for use in PoCT [22].

Colorimetric assays have been extensively used for the rapid detection of various diseases due to their simplicity, quick response, and high sensitivity [25,26,27,28,29,30,31]. Nanomaterials, especially gold nanoparticles (AuNPs), exhibit strong localized surface plasmon resonance (LSPR) properties which have been widely leveraged for the fabrication of colorimetric sensors [32]. AuNPs give off a colourful signal that can be visualized with the naked eye and without using advanced instruments [33]. Generally, a solution of colloidal AuNPs have a ruby-red colour due to their LSPR phenomenon that is highly dependent on interparticle distance [32,34,35]. Upon addition of salt, the colour of the AuNP solution changes from ruby red to purple/blue due to the shift of the LSPR to a higher wavelength. In contrast, when the AuNPs are bound to molecules, such as MRE for example, they will retain their ruby-red colour in the presence of salt. If the analyte for the specific MRE is present, the MRE binds to the analyte instead of the AuNPs and the colour of the AuNPs will change from ruby red to purple/blue. Based on this principle, several AuNP-based optical aptasensors have been developed [36,37,38,39,40]. In this study, using this principle, a simple yet effective, specific, and rapid RBP-A-aptasensor for the detection of RBP4 was developed. The assay is user-friendly as it does not require any specialised instruments and it is capable of producing a test result within 5 min, highlighting the potential applicability of this assay in PoCT and in resource-limited areas.

## 2. Materials and Methods

### 2.1. Materials

Retinol-binding protein aptamer (RBP-A) 5′-ATA CCA GCT TAT TCA ATT ACA GTA GTG AGG GGT CC GTC GTG GGG TAG TTG GGT CGT GGA GAT AGT AAG TGC AAT CT-3′ [21], was synthesized by Biomers.net GmbH (Ulm, Germany). RBP4, alpha-2-macroglobulin (A2MG), leptin and bovine serum albumin (BSA) were purchased from Sino Biologicals (Beijing, China), ProsPec (Ness-Ziona, Israel), R&D Systems (Minneapolis, MN, USA) and Merck (Pty) Ltd. (Rahway, NJ, USA), respectively. Chloroauric acid (HAuCl_4_·3H_2_O), sodium chloride (NaCl), and sodium citrate were purchased from Sigma-Aldrich (St Louis, MO, USA). Purified Milli-Q water was used in all experiments (Millipore, MA, USA). All the chemicals were of analytical grade.

### 2.2. Synthesis and Characterization of AuNPs

All glassware used for the preparation of AuNPs was first immersed thoroughly in aqua regia (1:3 (*v*/*v*) HNO_3_: HCl), then thoroughly washed with distilled water (dH_2_O) and dried overnight at 70 °C in an oven. AuNPs were prepared via the reduction of HAuCl_4_·3H_2_O using sodium citrate as described by Stiolica et al. [41,42]. The spectroscopic characterization of the synthesized AuNPs was carried out using Ultraviolet–visible (UV–vis) spectrophotometry (POLARstar Omega plate reader, BMG Labtech, Offenburg, Germany). The concentration of the AuNPs was evaluated using the UV–vis spectra as described by Haiss et al. [43]. The size distribution and zeta potential (ζ- potential) characterization of the AuNPs were determined using a Malvern NanoZS90 Zetasizer (Malvern Instruments Ltd., Malvern, UK) at a scattering angle of 90° at 25 °C. For the characterization of the size and morphology of the AuNPs, one drop of the sample solution was loaded onto a carbon-coated copper grid. The grids were dried for a few minutes under a Xenon lamp. High-resolution transmission electron microscope (HR-TEM) images were captured using an FEI Tecnai G^2^ 20 field-emission gun (FEG) HRTEM (Hillsboro, OR, USA) operated in bright field mode at an accelerating voltage of 200 kV.

### 2.3. Development of the RBP-A-Aptasensor

#### 2.3.1. Optimization of the Aptamer and NaCl Concentration

AuNPs are highly reactive and aggregate easily in the presence of salts; therefore, it is important to monitor the salt-induced AuNPs aggregation percentage for the development of a colorimetric aptasensor [44,45,46]. To optimize the performance of the developed assay, various conditions such as aptamer and NaCl concentrations were investigated. For this experiment, a total of 600 μL of different concentrations (0, 6.25, 12.5, 25, 50, 100 and 150 nM) of RBP-A was added to 1.08 mL of AuNPs in separate 2 mL Eppendorf tubes, mixed well, and incubated overnight at 25 °C. Then, 50 μL of the functionalized RBP-A-AuNPs (aptasensor) was added to a 96-well microtiter plate, containing 50 μL of dH_2_O. This was followed by the addition of different concentrations of NaCl (0, 20, 40, 60, 80 and 100 mM). The samples were incubated at 25 °C for 5 min and observed for colour change. The UV–vis spectra were measured using a plate reader, and the absorbance ratios (A650/A520) were calculated to determine the aggregation percentage of the AuNPs [47]. The experiment was performed in triplicate and the average absorbance ratios were calculated and used to plot bar graphs.

#### 2.3.2. RBP4 Detection Based on the Label-Free AuNPs-Based RBP-A-Aptasensor

For the detection of RBP4, 600 μL of 50 nM RBP-A and 1.08 mL of AuNPs were added to a 2 mL tube, mixed well, and incubated overnight at 25 °C. Subsequently, 50 μL of the aptasensor was added into the 96-well microtiter plate containing 25 µL of dH_2_O. Then, 25 μL of different concentrations of RBP4 (0, 7.8, 15.6, 31.2, 62.5, 125, 250 nM) were added into the aptasensor, mixed thoroughly, and incubated for another 5 min at 25 °C, and observed for colour change This was followed by the addition of 6 μL of 1 M NaCl solution (60 mM final concentration) and incubation at 25 °C for 5 min. The UV–vis spectra were measured using a plate reader and the absorbance ratios (A650/A520) were calculated to determine the aggregation percentage of the AuNPs. The experiment was performed in triplicate and the average absorbance ratios were calculated and used to plot the linear curve.

#### 2.3.3. Specificity of the Label-Free AuNPs-Based RBP-A-Aptasensor

To evaluate the specificity of the RBP-A-aptasensor, 50 μL of the aptasensor was added into the 96-well microtiter plate, containing 25 µL of dH_2_O. Then, 25 μL of 250 nM RBP4, A2MG, leptin and BSA were added into the aptasensor, mixed thoroughly, and incubated for another 5 min at 25 °C and observed for colour change. This was followed by the addition of 6 μL of 1 M NaCl solution (60 mM final concentration) and incubation at 25 °C for 5 min. A water sample was used as a blank. The UV–vis spectra were measured using a plate reader and the absorbance ratios (A650/A520) were calculated to determine the aggregation percentage of the AuNPs. The experiment was performed in triplicate and the average absorbance ratios were calculated and used to plot bar graphs.

## 3. Results and Discussion

AuNPs-based aptasensors have been widely applied in the detection of various targets including chemical compounds, proteins, pesticides, toxins, and viruses [36,38,39,40,48,49,50]. Their simple preparation, rapid detection, and the fact that they do not require special instrumentation have made them the preferred choice for PoCT [51]. The sensors rely on the salt-induced aggregation of bare AuNPs, leading to a change of colour from ruby red to purple/blue. In the absence of the target or biomarker molecule, the detecting molecule or MRE stabilizes the AuNPs against salt-induced aggregation and no colour change is observed [52]. Similarly, this study investigated the feasibility of the RBP-A-aptasensor for detection of RBP4. Although at least two other studies demonstrated the development of aptasensors for RBP4, neither of these assays can be used at the PoCT as both of these assays require specialised instruments and must therefore be carried out in a laboratory setting. In contrast, the assay described herein does not require any specialised instruments and can be carried out in the field.

### 3.1. Characterization of the AuNPs

**Figure 1:**
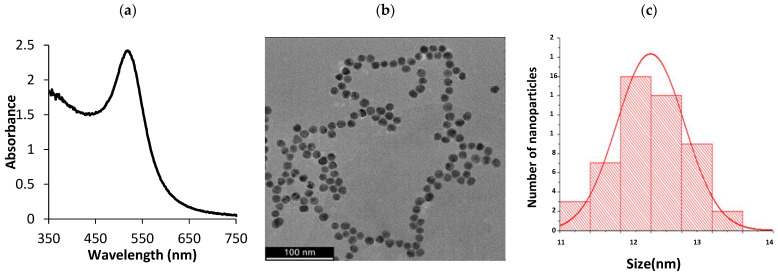
Characterization of AuNPs. (**a**) UV–vis spectrum of AuNPs; (**b**) a representative HR-TEM photomicrograph and (**c**) size distribution.

The synthesized AuNPs had an SPR peak at 519 nm (Figure 1a), indicating that the AuNPs were approximately 14 nm in size. The narrow peak suggested that the majority of the AuNPs were uniform and monodispersed [53]. The AuNPs had an absorbance value of 2.422 OD, and a concentration of 9.83 nM. DLS results revealed that the AuNPs had an average hydrodynamic diameter of 14.69 nm and a PDI of 0.175, the latter confirmed that the AuNPs were mostly monodispersed. The AuNPs showed a ζ- potential of −27.7 mV, indicating that the AuNPs were highly stable. TEM images revealed that the AuNPs were mostly spherical in shape and were relatively monodispersed (Figure 1b). This is due to electrostatic stabilization with a negatively charged layer of citrate ions [44]. The core diameters of AuNPs were found to be 13.98 ± 1.19 nm (Figure 1c), further corroborating the results obtained from the UV–vis spectrophotometry. The SPR results obtained in this study were similar to the results obtained by Stiolica et al., whereby 14 nm AuNPs also had an SPR of 519 nm. In contrast, the reported AuNPs had a ζ- potential of –51 mV [41], which was lower than the a ζ- potential obtained in this study.

### 3.2. Principles of AuNPs-Based RBP-A-Aptasensor

**Figure 2:**
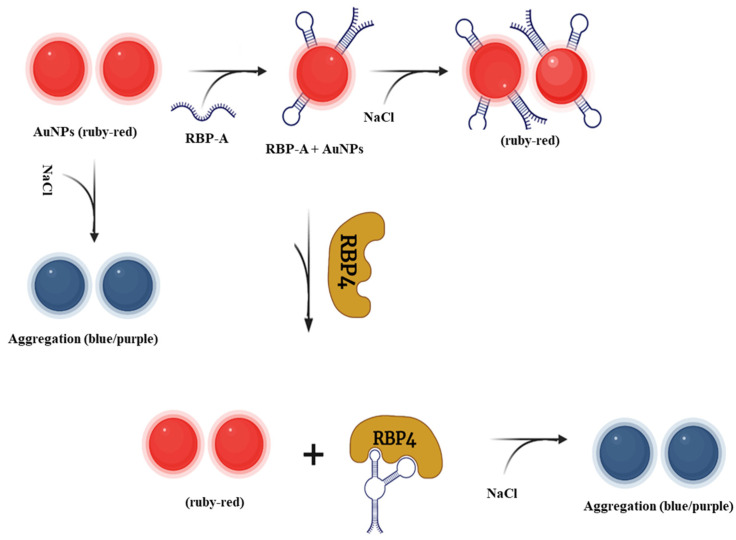
Schematic illustration of the colorimetric aptasensor for the detection of RBP4.

The principles of the AuNPs-based RBP-A-aptasensor for the detection of RBP4 is illustrated in Figure 2. Colloidal AuNPs produce a ruby-red colour in solution that is highly dependent on the interparticle distance [32,34,35]. In the presence of NaCl, the AuNPs undergo aggregation, leading to a visible colour change from ruby red to purple/blue. The presence of salt electrolytes decreased the interparticle distance of the AuNPs, thus reducing the ζ- potential and enabling electromagnetic interaction among the negatively charged AuNPs consequently leading to visible colour change [46]. In contrast, binding of RBP-A on the surface of AuNPs stabilized the AuNPs and prevented the NaCl-induced aggregation of the AuNPs [54,55]. The solution retained the ruby-red colour even at high NaCl concentrations (up to 60 mM). However, in the presence of RBP4, owing to the high affinity between RBP4 and RBP-A, RBP-A detached from the AuNPs and left the AuNPs unprotected. The bare AuNPs aggregated in the presence of NaCl, which led to a visible colour change from ruby red to purple/blue.

**Figure 3:**
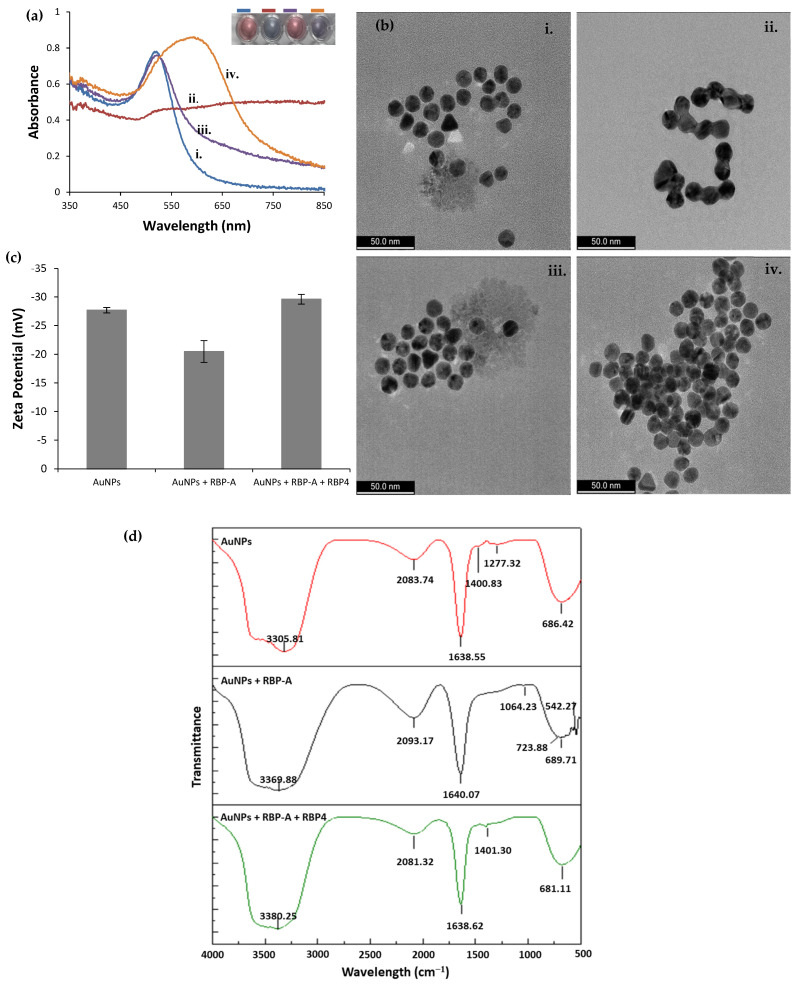
(**a**) UV–vis spectra, (**b**) HR-TEM of (i) AuNPs, (ii) AuNPs + NaCl, (iii) AuNPs + RBP-A + NaCl and (iv) AuNPs + RBP-A + RBP4 + NaCl, (**c**) ζ- potential and (**d**) FTIR analysis of the interaction between the RBP-A-aptasensor and RBP4. RBP4 was used at a concentration of 250 nM.

The compatibility of the AuNPs-based RBP-A-aptasensor for the detection of RBP4 was validated using four techniques: UV–vis spectroscopy, TEM, ζ- potential, and FTIR. As shown in Figure 3a, the AuNPs had an SPR peak at 519 nm which shifted to 560 nm upon the addition of NaCl, indicating aggregation. In the presence of RBP-A, the AuNPs were strongly protected against NaCl-induced aggregation and the SPR peak remained inert at 519 nm. Upon the addition of NaCl, the SPR peak shifted to 600 nm as a result of aggregation. This could be because, in the presence of RBP4, RBP-A detached from the AuNPs’ surface and bound to RBP4, leaving the AuNPs’ surface unprotected against salt-induced aggregation. HR-TEM was used to confirm these observations (Figure 3b). Bare AuNPs were monodispersed in the solution; however, in the presence of NaCl, the AuNPs aggregated and clumped together. The aptasensor without RBP4 showed no aggregation in the presence of NaCl, but in the presence of RBP4 and NaCl, the AuNPs aggregated.

The ζ- potential was also measured to monitor the changes on the AuNPs’ surface during the development of the aptasensor and when the aptasensor was incubated with RBP4 (Figure 3c). AuNPs exhibited a ζ- potential of −27.7 mV, which is in agreement with previously published data [56]. In contrast, the ζ- potential of −20.5 mV was obtained when RBP-A was incubated with the AuNPs solution, indicating that negative charges are partially neutralized by the aptamer covering the surface of the particles [47]. Finally, when RBP4 was added, the ζ- potential was restored to −29.6 mV, indicating that the RBP-A bound to RBP4 and left the AuNPs naked, thus returning them to their original ζ- potential. Using the same technique, Lerga et al. developed a AuNPs-based aptasensor for the detection of histamine. In their study, AuNPs exhibited a ζ- potential of − 47.6 mV which decreased to − 28.6 mV upon incubation of the aptamer with the AuNPs. The ζ- potential was restored to approximately − 50 mV after the addition of histamine [47].

Finally, FTIR (PerkinElmer Spectrum One FTIR spectrophotometer) analysis was carried out to monitor elemental changes to the AuNPs during the development of the aptasensor and when the aptasensor was incubated with RBP4 (Figure 3d). The FTIR spectrum of AuNPs features characteristic peaks at 3305.81, 2082.74, 1638.55, 1400.83, 1277.32 and 686.42 cm^−1^. The peak at 3305.81 cm^−1^ is attributed to the stretching vibration of OH and NH2. The peaks at 1400.82 cm^−1^ and 1638.55 cm^−1^ are attributed to the carboxylate symmetric and asymmetric stretching bonds of the carboxylate group (COO−) in citrate ions [57], respectively. The peak at 2083.74 cm^−1^ is attributed to the S-H group on the AuNPs [58], further validating successful synthesis of the AuNPs. Upon adsorption of the RBP-A, two weak peaks (1064.23 and 723.88 cm^−1^) were observed in RBP-A-AuNPs conjugate spectra which were absent in the spectrum for the AuNPs. The peak at 1064.23 cm^−1^ is attributed to the symmetric C=O stretching band of the phosphate backbone and the peak at 723.88 cm^−1^ is attributed to the C–H out of plane base vibration of the RBP-A [59], indicating successful adsorption of RBP-A on the surface of the AuNPs. Finally, when RBP4 is added, the peak attributed to the carboxylate asymmetric stretching band appeared at 1460.05 cm^−1^. This peak was also visible in the AuNPs, further supporting the assertion that RBP-A was bound to RBP4 and detached from the AuNPs.

### 3.3. Determination of the Optimum Concentration of NaCl and Aptamer

**Figure 4:**
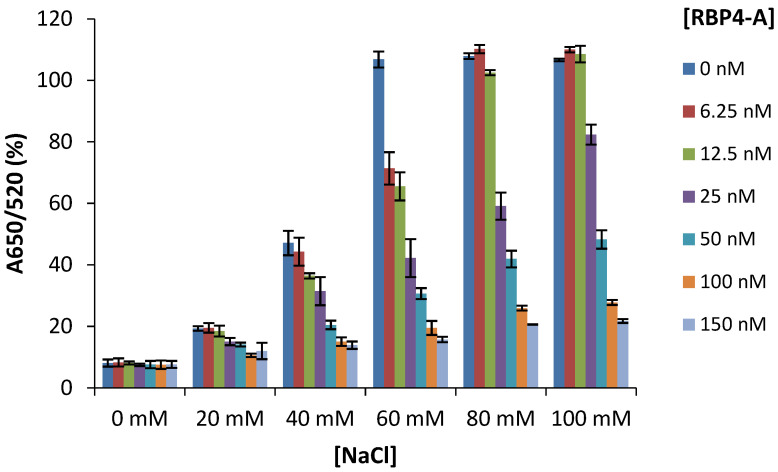
Stability of the AuNPs and RBP-A-aptasensor in the presence of increasing concentrations of NaCl. Final aptamer concentrations: 0, 6.25, 12.5, 25, 50, 100 and 150 nM; final NaCl concentrations: 0, 20, 40, 60, 80 and 100 mM.

The flocculation assay was used to determine the stability of the AuNPs-based RBP-A-aptasensor in the presence of different concentrations of NaCl. From Figure 4, it can be deduced that AuNPs and the RBP-A-AuNPs conjugates at all RBP-A concentrations showed no aggregation in the absence of NaCl. The AuNPs and the RBP-A-AuNPs conjugates at all RBP-A concentrations showed minimal aggregation below 20 mM NaCl; whereas, some aggregation was observed at 40 mM NaCl. The minimal changes of the absorbance at low NaCl concentrations were due to the low ionic strength of the electrolyte. At these NaCl concentrations, the aggregation of AuNPs is slow and the mean size of the formed aggregate is close to the size of the original AuNPs, rendering the changes insignificant. However, the AuNPs and the RBP-A-AuNPs conjugate solutions at lower RBP-A concentrations (6.25–12.5 nM) changed colour from ruby red to purple/blue with increasing concentrations of NaCl (60–100 mM), with the AuNPs reaching saturation levels between 60 and 100 mM NaCl. This observation suggested that the Na^+^ and Cl^−^ ions destroyed the ionic environment and led to the aggregation of AuNPs in the absence of aptamers [60]. In contrast, higher concentrations of RBP-A (25–150 nM) showed excellent protection efficiency against NaCl-induced aggregation of AuNPs, which was indicated by the retention of a ruby-red colour and a lower aggregation percentage. Overall, the results indicated that the addition of RBP-A caused a dose-dependent protection against NaCl-induced aggregation for all concentrations of NaCl. To ensure the assay is more cost effective, the optimal concentrations of RBP-A and NaCl for the development of the aptasensor were selected as 50 nM and 60 mM, respectively.

### 3.4. Sensitivity of the Aptasensor for RBP4 Detection

**Figure 5:**
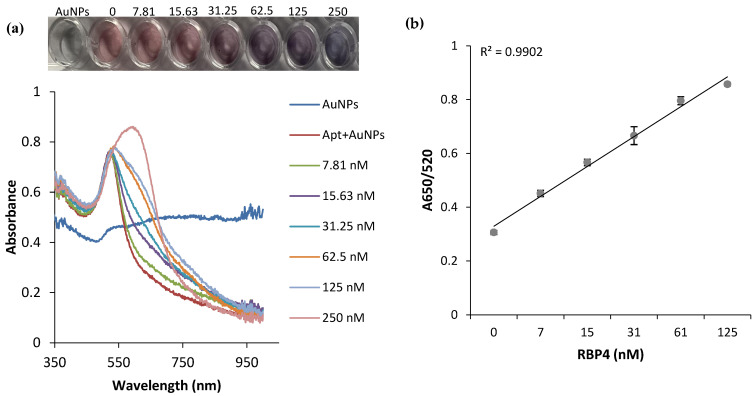
Analysis of the sensitivity of the AuNP-based colorimetric aptasensor. (**a**) Absorption spectra of RBP-A-AuNPs-aptasensor in the presence of increasing concentrations of RBP4. Insert: visual colour changes of the aptasensor incubated with increasing concentrations of RBP4 (final RBP4 concentrations: 0, 7.81, 15.63, 31.25, 62.5, 125 and 250 nM). (**b**) Linear calibration curve for different absorbance ratios (A620/A520) corresponding to different concentrations of RBP4 (0–125 nM).

The sensitivity of the AuNPs-based RBP-A-aptasensor for the detection of RBP4, under the optimized experimental conditions (50 nM RBP-A and 60 mM NaCl), was assessed by incubation with different concentrations of RBP4 and by calculating the absorbance ratios (A650/A520) to evaluate the aggregation of the AuNPs. Figure 5a and the insert indicate that the addition of NaCl caused a dose-dependent aggregation of the AuNPs, which is indicated by the gradual colour change of the AuNPs from ruby red to purple/blue and the shift in SPR from 519 to 600 nm. The results demonstrate that RBP-A bound to RBP4; thus, detaching from the AuNPs and leaving the AuNPs unprotected. In the presence of 60 mM NaCl, the AuNPs aggregated in a dose-dependent manner, with a more visible colour change between 62.5 and 250 nM. The assay was sensitive with a limit of detection (LOD) of 90.76 ± 2.81 nM. Moreover, the assay was rapid, and the test results were visually detectable within 5 min. The LOD was calculated as described by the International Union of Pure and Applied Chemistry (IUPAC) [44] as follows:

LOD = 3 × SD/S

where SD is the standard deviation of the response (y-intercept of the regression line) and S is the slope of the calibration curve.

### 3.5. Validation of the Aptasensor

Over the years, there has been a greater focus on the development of colorimetric aptasensors due to their simplicity, which is based on the visual detection of a test result. However, they frequently have sensitivity and reproducibility issues; thus, the reproducibility of the RBP-A-aptasensor was evaluated. The assay was performed in triplicate (at a concentration of 250 nM), on three separate days. As shown in Table 1, the recovery rate of RBP4 was in the range from 90.36 ± 3.60% to 101.49 ± 1.9% and the intra-assay coefficients of variation (CV) calculated ranged from 1,88% to 3,99%. The results for the RBP-A-aptasensor were comparable with the results of a commercially available Human ELISA kit (ab108897) which has a recovery rate of 97% and an intra-assay CV of 4.8%. In contrast, our RPB-A-aptasensor had an intra-assay CV of 3.12%, which is better than that of the ELISA kit (8.5%), further confirming the exceptional reproducibility of the RBP-A-aptasensor.

### 3.6. Specificity of the Aptasensor for RBP4

**Figure 6:**
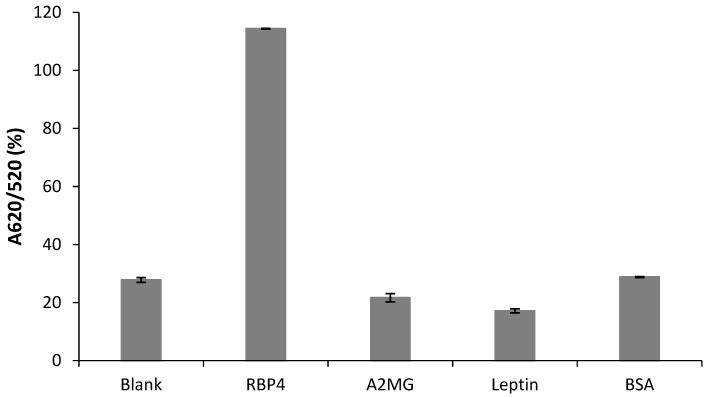
Specificity of the RBA-A-aptasensor for RBP4 detection. The specificity experiment was performed at a concentration of 250 nM for all proteins. The blank sample contained water.

The specificity of the developed RBP-A-aptasensor for the detection of RBP4 was evaluated against two diabetes-related biomarkers (A2MG and leptin) and one serum-abundant protein (BSA). The proteins (at a concentration of 250 nM) were individually incubated with the RBP-A-aptasensor, and the absorbance ratios of A620/A520 were calculated (Figure 6). A water solution was used as the blank. The RBP-A-aptasensor showed a colour change from ruby red to purple/blue in the presence of RBP4 and no colour change was observed in the presence of A2MG, leptin and BSA. This indicated that RBP4 had a very high response to the RBP-A-aptasensor; whereas, A2MG, leptin and BSA displayed no interaction with the RBP-A-aptasensor. Previous studies have also evaluated the specificity of RBP-A towards RBP4 using various proteins such as RBP4, adiponectin, vaspin, nampt, BSA, human serum albumin, human IgG, fibrinogen, insulin and anti-RBP4 antibody and no interference was detected [21,23], further corroborating our results that RBP-A is highly specific to RBP4.

### 3.7. Comparison of the Aptasensor with Other Aptamer-Based Approaches for RBP4 Detection

Since the identification of RBP-A in 2008 by Lee et al. [36], there have been only three studies published detailing aptamer-based assays for the detection of RBP4. A comparison of the results of these assays is provided in Table 2. The LODs for ELAAS, SPR and chemiluminescence were 3.39 nM, 75 nM, and 0.04 pM, respectively [36,53,54]. In contrast, the aptasensor developed in this study had a limit of detection of 90.76 nM. This indicated that the developed aptasensor was less sensitive when compared to the aptamer-based SPR, ELAAS and chemiluminescence sensors. Nevertheless, the results in this study were still within the normal detection range. More importantly, the results for this assay were obtained within 5 min, whereas the results for the other assays were obtained after approximately 2 h, indicating that the developed aptasensor was more rapid. Additionally, confirmation of the test results is based on visual detection of colour changes without the need for any advanced instruments, a laboratory or a power source. This further indicates that the aptasensor is suitable for PoCT.

**Figure 7:**
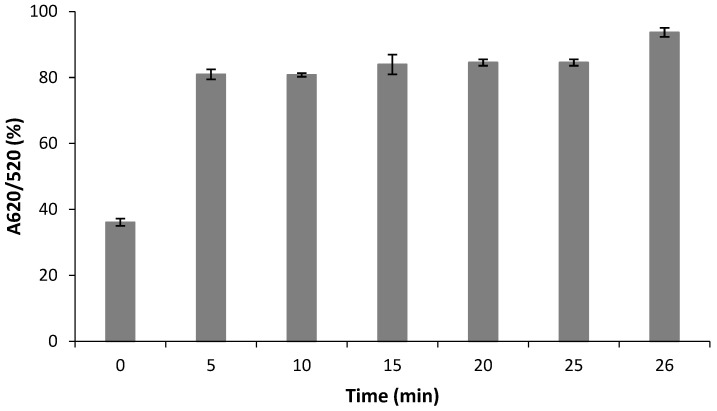
Aggregation of the RBP-A-aptasensor in the presence of 60 mM NaCl at various incubation periods.

The incubation time of the aptasensor with RBP4 was also evaluated. This was carried out by incubating RBP4 (at a concentration of 250 nM) with the aptasensor for different time periods (0–30 min), followed by the UV–vis measurements and calculation of the absorbance ratios (A620/A520) to determine the degree of aggregation of the AuNPs (Figure 7). The results indicated that the aptasensor had lower aggregation in the presence of NaCl, as indicated by the lower absorbance ratio. When the aptasensor was incubated with RBP4 for 5 to 30 min, followed by the addition of NaCl, the aggregation increased and reached saturation levels between 5 and 30 min, indicating that 5 min incubation of the aptasensor and RBP4 was sufficient for binding. The interaction of RBP-A with RBP4 was further analyzed using the MicroScale Thermophoresis (MST) technique (Monolith NT.115, Nanotemper). As shown in Table 3, MST revealed that RBP-A bound with a dissociation constant (Kd) of 3.523 nM within 1.5 s and continued until it reached a Kd of 13.438 nM within 20 s. This is attributed to the high affinity of the aptamer for the target thus allowing rapid recognition. One of the major advantages of PoCT is that it provides much faster access to test results, allowing for more rapid clinical decision-making and more appropriate treatments and interventions. This means that standard RBP4 detection assays such as ELISAs, which require longer incubation periods, may be replaced by the aptasensor at the PoC, allowing the process from sample collection to data analysis to happen within 10 min.

## 4. Conclusions

In this study, a label-free colorimetric aptasensor for the detection of RBP4 using ssDNA aptamer and unmodified AuNPs was successfully developed. The assay is based on colour change from ruby red to blue/purple due to the binding of RBP-A to RBP4, which leaves AuNPs exposed to the phenomenon of salt-induced AuNP aggregation. The assay was sensitive, with an LOD of 90.76 ± 2.81 nM. Further development using clinical samples on non-diabetic and diabetic patients is required to assess the utility of the aptasensor for the early detection of T2DM.

## Figures and Tables

**Table 1:** Mean Recoveries and Coefficients of Variation for RBP4.

Day	Mean Recovery ± SD (%)	CV (%)	Avarage CV (%)
1	90.36 ± 3.60	3.99	3.12
2	94.95 ± 3.13	3.48	
3	101.49 ± 1.91	1.88	

SD: Standard deviation.

**Table 2:** Comparison of the RBP-A-aptasensor with other methods used for RBP4 detection.

MRE	Method	Nanoparticles	LOD	Detection Time	Reference
Aptamer	SPR	None	75 nM	2 h and 20 min	[21]
Aptamer and antibodies	ELAAS	None	3.39 nM	2 h	[23]
Aptamer and antibodies	Chemiluminescence	AuNPs	0.04 pM	2 h	[24]
Aptamer	Colorimetric	AuNPs	90.76 nM	5 min	Present study

**Table 3:** MST analysis of the interaction of RBP-A with RBP4.

Time (s)	1.5	2.5	5	10	15	20
Kd (nM)	3.523	4.6723	6.6014	8.313	11.501	13.438

## Data Availability

The data generated in this study has been represented as Tables and Figures in the manuscript and available from the corresponding author upon request.
